# StreptomeDB 4.0: a comprehensive database of streptomycetes natural products enriched with protein interactions and interactive spectral visualization

**DOI:** 10.1093/nar/gkae1030

**Published:** 2024-11-05

**Authors:** Yue Feng, Ammar Qaseem, Aurélien F A Moumbock, Shuling Pan, Pascal A Kirchner, Conrad V Simoben, Yvette I Malange, Smith B Babiaka, Mingjie Gao, Stefan Günther

**Affiliations:** Institute of Pharmaceutical Sciences, Albert-Ludwigs-Universität Freiburg, Hermann-Herder-Str. 9, D-79104 Freiburg, Germany; Institute of Pharmaceutical Sciences, Albert-Ludwigs-Universität Freiburg, Hermann-Herder-Str. 9, D-79104 Freiburg, Germany; Institute of Pharmaceutical Sciences, Albert-Ludwigs-Universität Freiburg, Hermann-Herder-Str. 9, D-79104 Freiburg, Germany; Institute of Pharmaceutical Sciences, Albert-Ludwigs-Universität Freiburg, Hermann-Herder-Str. 9, D-79104 Freiburg, Germany; Institute of Pharmaceutical Sciences, Albert-Ludwigs-Universität Freiburg, Hermann-Herder-Str. 9, D-79104 Freiburg, Germany; Structural Genomics Consortium, University of Toronto, 101 College Street, Toronto, ON M5G 1L7, Canada; Research Unit in Nutrition, Health, Functional Foods and Nutraceuticals, Universidad San Ignacio de Loyola, Av. La Fontana 550, Lima PE-15024, Peru; Department of Chemistry, University of Buea, Molyko, PO Box 63, Buea, Cameroon; Department of Microbial Bioactive Compounds, Eberhard Karls Universität Tübingen, Auf der Morgenstelle 28, D-72076 Tübingen, Germany; Weifang People’s Hospital, Shandong Second Medical University, 151 Guangwen St, Weifang 261041, China; Institute of Pharmaceutical Sciences, Albert-Ludwigs-Universität Freiburg, Hermann-Herder-Str. 9, D-79104 Freiburg, Germany

## Abstract

Streptomycetes remain an important bacterial source of natural products (NPs) with significant therapeutic promise, particularly in the fight against antimicrobial resistance. Herein, we present StreptomeDB 4.0, a substantial update of the database that includes expanded content and several new features. Currently, StreptomeDB 4.0 contains over 8500 NPs originating from ∼3900 streptomycetes, manually annotated from ∼7600 PubMed-indexed peer-reviewed articles. The database was enhanced by two in-house developments: (i) automated literature-mined NP–protein relationships (hyperlinked to the CPRiL web server) and (ii) pharmacophore-based NP–protein interactions (predicted with the ePharmaLib dataset). Moreover, genome mining was supplemented through hyperlinks to the widely used antiSMASH database. To facilitate NP structural dereplication, interactive visualization tools were implemented, namely the JSpecView applet and plotly.js charting library for predicted nuclear magnetic resonance and mass spectrometry spectral data, respectively. Furthermore, both the backend database and the frontend web interface were redesigned, and several software packages, including PostgreSQL and Django, were updated to the latest versions. Overall, this comprehensive database serves as a vital resource for researchers seeking to delve into the metabolic intricacies of streptomycetes and discover novel therapeutics, notably antimicrobial agents. StreptomeDB is publicly accessible at https://www.pharmbioinf.uni-freiburg.de/streptomedb.

## Graphical Abstract

**Graphical Abstract:**
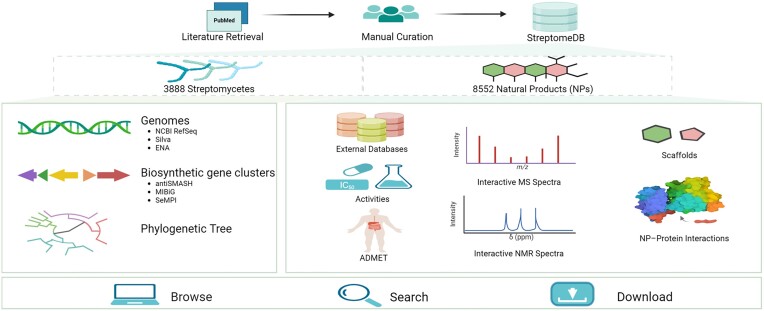

## Introduction

Streptomycetes (bacteria of the genus *Streptomyces*) have long been recognized as an unparalleled source of bioactive natural products (NPs), contributing significantly to the pharmaceutical arsenal against various diseases (1,2). These Gram-positive filamentous bacteria are widely distributed in terrestrial, estuarine and marine ecosystems. They exhibit remarkable adaptability to extreme environments and possess a diverse array of strains, each harbouring multiple biosynthetic gene clusters (BGCs) (3–6). Owing to these unique biological characteristics, streptomycetes synthesize a broad spectrum of NPs with diverse scaffolds and biological activities. Hence, streptomycetes are well positioned as an invaluable resource for discovering novel therapeutic agents, especially in response to the rising antimicrobial resistance (7,8).

The urgency of exploring the still largely untapped reservoir of streptomycetes NPs prompted the development of StreptomeDB (9), which was first launched in 2012. Over the years, the database has been incrementally enhanced in subsequent releases (10,11), with newly annotated NPs, source organisms and features to explore their biosynthetic origins and bioactivities. StreptomeDB is widely used in the scientific community to facilitate the exploration of streptomycetes NPs, notably for structural dereplication. For instance, Das *et al.* (12) utilized the database to dereplicate cinnabaramide A, a covalent inhibitor of the human 20S proteasome isolated from *Streptomyces murinus* THV12, by matching the experimental liquid chromatography–electrospray ionization tandem mass spectrometry (LC/ESI-MS/MS) data with predicted MS spectra in StreptomeDB. Similarly, Nogami *et al.* (13) applied this utility to dereplicate cycloheximide, an inhibitor of seed germination in *Orobanche minor*, by comparing experimental ESI-MS spectra with the database’s predicted MS data. Beyond dereplication, StreptomeDB has been instrumental in metabolite annotation. For instance, Wang *et al.* (14) employed StreptomeDB NPs to confirm the identities of metabolites whose production increased following the integration of the pyrroloquinoline quinone BGC into various *Streptomyces* strains. Additionally, StreptomeDB has been used for structure-based virtual screening. For instance, Macalalad *et al.* (15) computationally docked all StreptomeDB NPs against a crystal structure of the Nipah virus matrix protein, leading to the identification of nargenicin A1 as a potential inhibitor.

StreptomeDB 4.0 aims to enhance the depth and breadth of information available to users. Several new features have been incorporated, including literature-mined NP–protein relationships as well as pharmacophore-based predictions of NP–protein interactions, facilitating studies of mechanisms of action and target-based drug discovery. Additionally, the introduction of interactive visualization for predicted nuclear magnetic resonance (NMR) and MS significantly enhances the database’s utility for structural dereplication.

## Growth of the database

This release integrates data from peer-reviewed PubMed-indexed articles published over the last 4 years. Initially, all PubMed abstracts containing either the word ‘streptomycetes’ or ‘Streptomyces’ were programmatically retrieved with NCBI Entrez (16). Next, entities (compounds and species) were tagged using PubTator (17), and only articles containing an entity pair were retained. Finally, the resulting dataset was manually curated for accuracy and completeness. The current contents of the database are summarized in Table 1. This release includes the addition of 2028 NPs, bringing the total to 8552 NPs, along with a notable increase in the total number of unique scaffolds to 7793. The database now features 3888 organisms, encompassing a diverse array of strains. The relationships between NPs and organisms have grown to an extensive 14 172, while NP–biosynthesis route relationships have reached 1928. The interactive phylogenetic exploration of organisms and their NPs is facilitated through an integrated phylogenetic tree, as established in previous releases (10). Furthermore, adding predictive NMR, MS and ADMET (absorption, distribution, metabolism, excretion and toxicity) data for an expanded number of NPs significantly increases their utility for users. For the first time, this version introduces 336 228 NP–protein relationships mined from the PubMed-indexed literature, as well as 398 717 predicted NP–protein interactions, expanding the database’s scope and potential for target-based drug discovery. These statistics not only highlight the growing enthusiasm within the scientific community for isolating bioactive NPs, but also underscore the importance of StreptomeDB as an essential resource in the ongoing quest for novel therapeutics derived from streptomycetes.

**Table 1.** Statistics of StreptomeDB attributes across its releases

	Release number			
**Attribute**	**1**	**2**	**3**	**4**
Publication year	2012	2015	2020	2024
NPs	2444	4040	6524	8552
Unique scaffolds	–^a^	4680	6262	7793
Organisms (including strains)	1985	2584	3302	3888
NP–organism relationships	4341	6717	10 912	14 172
NP–biosynthesis route relationships	307	731	1392	1928
NP–activity relationships	1036	3813	6850	8947
NPs with predicted NMR spectra	–	3989	6507	8551
NPs with predicted MS spectra	–	1945	4943	8520
NPs with predicted ADMET properties	–	–	6524	8287
Referenced articles	4544	5486	6754	7630
NP–protein relationships in the literature	–	–	–	336 228
Predicted NP–protein interactions	–	–	–	398 717

^a^Not yet implemented.

## Recent developments

### Literature-mined NP–protein relationships

In this release, literature-mined NP–protein relationships were introduced as an innovative feature, through hyperlinks to the Compound–Protein Relationships in Literature (CPRiL) web server, which we recently developed (18). Conceptually, an NP–protein relationship denotes a functional association in which an NP and a protein interact directly, regulate each other or are integral parts of one another (19). In CPRiL, molecular entities (compounds and genes/proteins) mentioned in PubMed abstracts are annotated using PubTator (17). A fine-tuned BioBERT machine learning model (20) was then employed to uncover relationships between entity pairs based on their co-occurrence within sentences of the articles, typically identified by interaction verbs. The confidence of the mined relationship is based on the performance of the model, which has a precision of 82.9%, a recall of 85.7% and an F1 score of 84.3%. Finally, streptomycetes NPs were mapped to CPRiL entries via the compound name and synonyms. In total, 336 228 NP–protein relationships are documented in CPRiL for all StreptomeDB entries. Hyperlinks to CPRiL direct users to a network display of these relationships based on their frequency in biomedical literature. This feature enables deeper insights into the mechanisms of action of these bioactive NPs.

### Pharmacophore-based predictions of NP–protein interactions

While numerous NP–protein relationships have been documented in the literature, most focus on well-characterized streptomycetes NPs that were isolated many years ago, e.g. staurosporine, a pan-kinase inhibitor. In contrast, the mechanisms of action for NPs isolated in recent years remain largely unknown, highlighting the potential of computational methods to predict these mechanisms prior to experimental validation (21,22). In this context, we used the in-house ePharmaLib dataset (23), which contains 15 148 therapeutically relevant e-pharmacophores (labelled as ‘PDBID-hetID-UniprotEntryName’), to predict potential target proteins for each NP in StreptomeDB. Specifically, a 3D conformer dataset for each streptomycetes NP was generated using LigPrep (Schrödinger LLC, New York, USA) and RDKit (24). Then, they were rigidly aligned onto all e-pharmacophores in parallel using Align-it (25) and GNU parallel (26). The predicted interactions are ranked based on a metric (0 ≤ Tverskyscore ≤ 1), indicating their likelihood of occurrence. Only statistically significant interactions with a likelihood of at least 70% (i.e. Tverskyscore ≥ 0.7) were retained, resulting in 399 136 NP–protein interactions. The effectiveness of ePharmaLib has previously been demonstrated through a retrospective evaluation with staurosporine (hetID: STU), whereby a substantial proportion of the top-ranked predictions corresponded to established NP–protein interactions (23). Therefore, the integrated ePharmaLib predictions could assist in mechanism of action studies and target-based drug discovery. Nevertheless, due to the inherent limitations of rigid pharmacophore alignments in accurately mimicking molecular recognition events, flexible molecular docking, molecular (meta)dynamics and/or free energy calculations of the predicted NP–protein interactions are warranted prior to experimental validation.

### Biosynthetic gene clusters

In previous releases, StreptomeDB provided hyperlinks to experimentally characterized BGCs associated with streptomycetes NPs via MIBiG (27) and predicted NPs linked to SeMPI (28). In the current release, we have enhanced the genome mining capabilities of StreptomeDB by incorporating hyperlinks to predicted BGCs from the antiSMASH database (29), mapped to retrieved NCBI Reference Sequence accession numbers (30). The antiSMASH database is the largest of its kind, currently hosting 231 534 high-quality BGCs from 35 726 bacterial genomes and supporting 88 distinct biosynthetic pathway types (29). By leveraging the combined data from StreptomeDB and antiSMASH, researchers are better equipped to explore opportunities in NP production and mutasynthesis, ultimately enhancing the pursuit of new therapeutic agents.

### Interactive spectral visualization

**Figure 1.**
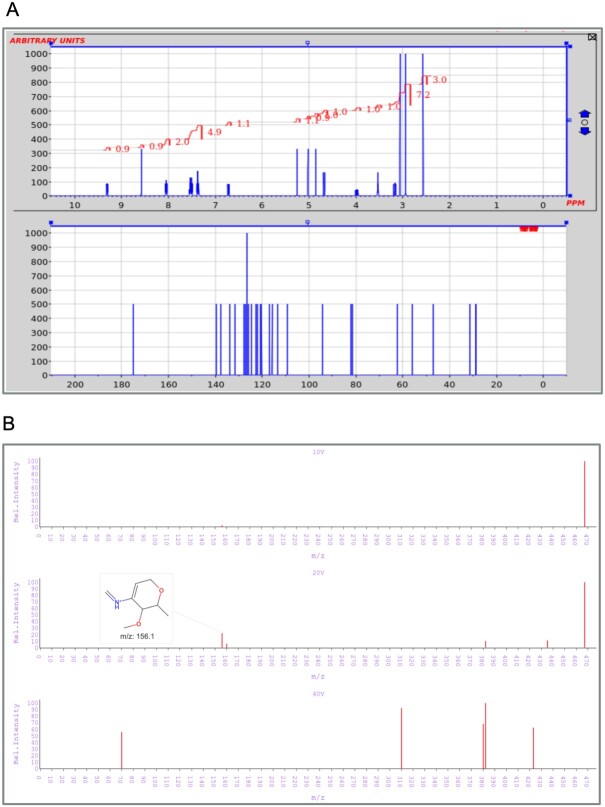
Interactive spectral plots of staurosporine. (**A**) Stacked ^1^H/^13^C NMR plots, with peak integration for ^1^H NMR. (**B**) Stacked 10 V/20 V/40 V MS plots, displaying the structure of a fragment corresponding to a peak in the 20 V MS spectrum.

To optimize performance and improve the user experience, both the backend database and the frontend web interface were redesigned, and several software packages, including PostgreSQL and Django, were updated to the latest versions. To facilitate NP structural dereplication, interactive visualization tools have now been implemented, namely the JSpecView (31) applet and plotly.js (https://plotly.com/javascript/) charting library for predicted NMR JCAMP-DX files and MS TXT files, respectively, which have so far been represented as tables in previous versions. These NMR and MS data were generated using the command-line tools CFM-ID (32) and cxcalc (Marvin 23.16.0, ChemAxon, https://chemaxon.com/), respectively. Figure 1 presents example spectral plots for staurosporine. The user can toggle between ^1^H, ^13^C or stacked ^1^H/^13^C NMR spectral plots (Figure 1A) and zoom in on a specific area of interest. For MS, the user can hover over a peak to see the corresponding *m*/*z* value and structure of the predicted fragment on one of three stacked 10 V/20 V/40 V spectral plots (Figure 1B). These interactive plots could enable users to easily compare with experimental results, thereby simplifying the identification and characterization of isolated streptomycetes NPs. It is worth mentioning that StreptomeDB also offers hyperlinks to experimental NMR and MS spectra available for some entries through NMRShiftDB (33) and GNPS (34), respectively.

## Conclusions

The latest release of StreptomeDB features an extensive collection of 8552 unique NPs sourced from 3888 streptomycetes. The interactive phylogenetic exploration of these organisms and their NPs is facilitated through an integrated phylogenetic tree. Moreover, hyperlinks to the antiSMASH database provide access to predicted BGCs, offering essential insights into the genetic context that guides further research on NP production and mutasynthesis. By integrating literature-mined data and predicted protein interactions alongside interactive spectral visualization, StreptomeDB 4.0 could aid researchers in understanding the biological mechanisms of these NPs. Overall, this database serves as a vital resource for researchers investigating the metabolic intricacies of streptomycetes and potentially discovering novel therapeutics to combat the growing global health threat posed by antimicrobial resistance. Future updates will focus on expanding the dataset and enhancing predictive capabilities.

## Data Availability

StreptomeDB is publicly accessible at https://www.pharmbioinf.uni-freiburg.de/streptomedb/. Its compounds and associated metadata are available for download as a single SDF file.
